# Lupus Pneumonitis Therapy Masks Coronavirus (COVID-19)

**DOI:** 10.1155/2021/6645780

**Published:** 2021-04-08

**Authors:** S. Soloway, N. L. DePace, A. M. Soloway, J. Colombo

**Affiliations:** ^1^Chairman Division of Rheumatology, Department of Medicine, Inspira Health Network, Vineland, NJ, USA; ^2^President Arthritis & Rheumatology Associates of South Jersey PC, Vineland, NJ, USA; ^3^Drexel University College of Medicine, Vineland, NJ, USA; ^4^Rowan University School of Osteopathic Medicine, Vineland, NJ, USA; ^5^Franklin Cardiovascular Associates PA, Washington Township,, NJ, USA; ^6^Autonomic Dysfunction and POTS Center, Sewell, NJ, USA; ^7^Department of Medicine, Inspira Health Network, Vineland, NJ, USA; ^8^Parasympathetic & Sympathetic Nervous System Consultant, Franklin Cardiovascular Associates PA, Vineland, NJ, USA

## Abstract

**Introduction:**

Coronavirus pneumonitis can mimic, or present as, lupus pneumonitis. Lupus may cause inflammation of the myocardium. Lupus pneumonitis high-dose steroid therapy may mask coronavirus (COVID-19). *Case Study*. The patient is a 65 y/o Hispanic female with lupus pneumonitis complicated by COVID-19. Her diagnosis was confirmed by a renal biopsy. She had nephritic and nephrotic syndrome. She was hospitalized a month earlier with shortness of breath with pulmonary infiltrates and was treated with steroids. The symptoms resolved quickly with shrinking consolidations and infiltrates. The patient returned to the office for shortness of breath with a presumptive diagnosis of recurrent lupus pneumonitis from steroid taper. The patient had a CT scan of the chest that revealed upper lobe interstitial and peripheral infiltrates. The radiologist felt that this was suspicious for coronavirus pneumonitis, and the patient was placed in isolation and continued therapy for lupus pneumonitis. She deteriorated, became hypoxic, and ventilated.

**Conclusion:**

All lupus pneumonitis patients, in fact all lupus patients in general (personal experience), on high-dose steroid therapy should be tested for COVID-19 to ensure proper diagnosis and therapy planning.

## 1. Introduction

Pneumonitis is a feature of systemic lupus erythematosus (SLE or lupus). SLE is a systemic, multisystem disease characterized by antibodies directed against anti-double-stranded DNA. Lupus may cause inflammation of the myocardium. Its prevalence is higher in women (female-to-male ratio of 10 : 1), and approximately half of the patients will experience pulmonary involvement [1]. SLE is characterized by the production of pathogenic autoantibodies directed against nucleic acids and their binding proteins, reflecting a global loss of self-tolerance [2]. The loss of tolerance with subsequent immune dysregulation is a consequence of genetic factors, in the setting of environmental triggers and stochastic events [3]. The loss of immune tolerance, increased antigenic load, excess T-cell help, defective B-cell suppression, and the shifting of T helper 1 (Th1) to Th2 immune responses lead to B-cell hyperactivity and the production of pathogenic autoantibodies. SLE spares no organ system, and involvement occurs but is not limited to the following: joints, skin, kidneys, bone marrow, peripheral blood, brain, heart, and lungs. Symptoms vary but may include fatigue, pain, serositis, and glomerulonephritis [4, 5]. Pulmonary involvement in SLE includes lupus pneumonitis [4]. As a result, lupus pneumonitis mimics, or may present as, coronavirus (COVID-19 or SARS-CoV-2), specifically coronavirus pneumonitis. Patients with lupus that develop pneumonitis and fail to respond rapidly to conventional treatments should be regarded with a high index of suspicion for coronavirus.

Lupus pneumonitis is characterized by fever, dyspnea, chest pain, and cough with or without hemoptysis [6]. This is due to the inflammation of the lungs. Pulmonary involvement in lupus may include pleurisy, interstitial lung disease, pneumonitis, shrinking lung syndrome, pulmonary artery hypertension, and pulmonary embolus [4, 7]. Lungs may scar from the inflammation and cause worsening shortness of breath. While there is no cure for lupus, current treatments focus on improving quality of life through controlling symptoms and minimizing flare-ups [8], including sun protection and diet, with anti-inflammatories and short-term steroids [9, 10], and possibly immunosuppressive medications which may expedite the tapering or discontinuation of steroids and may prevent disease flares [11]. There is evidence to support the beneficial effects of B-cell targeting and biological agents [12, 13]. Anti-inflammatories and steroids are also treatments for coronavirus (COVID-19) [14]. When evaluating a lupus patient with shortness of breath, one must consider non-lupus-related conditions such as infections and viruses, including COVID. SLE patients are often immunocompromised due to disease or medications, and opportunistic infections should be considered, again, including coronavirus. COVID-19, a severe acute respiratory syndrome, is predominately an upper respiratory infection, and a large subset of patients may be asymptomatic [15], especially if they are already on steroids or other therapies that also treat COVID. Given the spread of the infection, it has also become imperative to consider this virus when beginning patients on immunosuppressive therapies.

## 2. Summary

This is a case of a 65-year-old Hispanic female with lupus pneumonitis complicated by COVID-19 infection. Her diagnosis was confirmed by a renal biopsy which revealed class IV and V glomerular lesions with full-house immunofluorescence. This patient had nephritic and nephrotic syndrome (30 g of protein in 24 hours). She was hospitalized a month earlier with shortness of breath with pulmonary infiltrates and was treated with high-dose steroids (1 mg/kg Solu-Medrol twice daily intravenously). The pulmonary symptoms resolved quickly. Her consolidations and infiltrates (lower lobes) were shrinking (they were seen at her initial hospitalization which helped lead to the diagnosis of lupus pneumonitis). The patient was discharged from the hospital on a tapering course of steroids. Forty-eight hours after discharge, the patient returned to the office with severe shortness of breath. The assumption was made that the patient was weaned too rapidly from steroids and in fact probably had recurrent lupus pneumonitis. However, readmitting the patient to the hospital and giving high-dose IV steroids did not help. Subsequently, within 24 hours, the patient was diagnosed with COVID pneumonia. These all occurred actually two or three days after discharge and weaning steroids. The course of weaning went from 80 mg to 60 mg to 40 mg to 20 mg over that time period. The patient had a CT scan of the chest that revealed new bilateral upper lobe interstitial infiltrates and scattered peripheral infiltrates. Due to the current pandemic, the patient was isolated and remained on hydroxychloroquine (the patient demonstrated normal EKG with normal QT-interval and was prescribed medications that also protect against the possible long-term effects of hydroxychloroquine), mycophenolate, and oral prednisone 60 mg daily for lupus pneumonitis. Clinically, the patient deteriorated, became hypoxic, and was mechanically ventilated. Likely based on simply being in the hospital or living in close quarters with many family members, she developed coronavirus and, at the time of this writing, has been sick in bed. The patient has provided written, informed consent to publish her case details. Coronavirus pneumonitis/pneumonic infiltrates can mimic, or as in this case present as, lupus pneumonitis. Furthermore, we now propose COVID-19 screening for lupus patients with shortness of breath or constitutional symptoms of unknown etiology.

## 3. Case Study

The patient is a 65-year-old Hispanic female, 5′ 1.5″, 213 lb. (BMI 39.6), and had a resting HR of 99 bpm, a resting BP of 119/85 mmHg, and a resting pain level (1–10) of best day, 3; worst day, 10; today, 8. She originally presented to an outpatient rheumatology clinic with anasarca. Her evaluation revealed greater than 20 g of protein in a 24-hour urine collection. Renal biopsy revealed membranous nephropathy (class V lesion) and diffuse proliferative glomerulonephritis (class IV lesion), and full-house immunofluorescence was noted. Class IV lesions indicate that over 50% of the glomeruli were involved. Full-house glomerulonephropathy is characterized by an immunofluorescence pattern of concomitant deposition of immunoglobulins—IgG, IgA, and IgM—and two complement system components—C3 and C1q—in renal tissue. This patient had nephritic syndrome and nephrotic syndrome. She presented with 15 g of protein in the urine and ended up being hospitalized.

She wound up leaving the hospital with 30 g of protein in the urine. However, in the hospital, she received pulse steroids and 1.2 g of cytoxan. She is on mycophenolate, 3 g daily, but experiencing loose stools. She was fully anticoagulated as her albumin had been under 2 and at that time was anticoagulated due to her nephrotic syndrome. She is on lipitor 80 mg daily and currently on metoprolol ER 50 mg, irbesartan 150 mg daily, and Bumex 2 mg daily, all for her hypertension and resolving anasarca.

At her first office visit since she was seen in the hospital, she presented with disproportionate lower extremity swelling. She reports her urine flow is adequate, she is very tired and weak, and today is the first day she has been out of the house. Her baseline blood studies prior to going to the hospital showed a hepatitis C exposure with a viral load of less than 1.18 million. Her ANA was 2560, homogeneous double-stranded DNA was 361, vitamin D_2_ level was 6 ng/dl, SSA was greater than 8, ESR was 101, Smith antibody was greater than 8, RNP antibody was greater than 8, urinalysis showed 3+ blood and 3+ protein, renal biopsy showed class IV and class V lupus nephritis, C4 was 11, C3 was 81, hemoglobin was 11, BUN and creatinine were 15/1.15, calcium was 7.9, albumin was 1.9, and GFR was 50.0. She received vancomycin and imipenem empirically, and pan cultures were negative. She did experience lupus pneumonitis. Concerns about lupus pneumonitis were disseminated to infectious disease, intensive care, and the hospitalist providing care for the patient but were ignored. There was a five-day delay in getting her cytoxan. Eventually, she did receive 1.2 g of cytoxan.

Upon presentation to the office, the patient complained of fatigue, insomnia, and malaise, gastroesophageal reflux, foamy urine and dark-colored urine, and diffuse joint swelling. The patient admits to being sun sensitive but denied easy bruising, nodules or bumps, and rash. She denied all other common indications. She was aware of being anemic. Physical exam revealed disproportionate bilateral 4+ bipedal and pretibial edema, and anasarca with no active synovitis. Current medications include prednisone (20 mg, tid), mycophenolate mofetil (1000 mg, bid), omeprazole (40 mg, qd, days), losartan (25 mg, qd, days), metoprolol succinate ER (50 mg, qd), irbesartan (150 mg), bumetanide (2 mg, qd), bumetanide (1 mg, qd), hydroxychloroquine (200 mg, bid), hydrochlorothiazide (25 mg, qd), ondansetron (4 mg, qd), atorvastatin (80 mg, qd), excedrin migraine (250 mg-250 mg-65 mg tablet, prn), and cholecalciferol (vitamin D_3_, 50000 meg, weekly).

Past medical history included abdominal surgery for endometrial ablation, cataract surgery, cholecystectomy performed in 2020, tubal ligation, and vascular surgery of the left leg. Her family history was negative for diabetes or hypertension and negative for lupus. She denies smoking, alcohol, or illicit drugs, and she had no known drug allergies.

Plan is for mycophenolate 2 g daily, as tolerated, based on her kidney function and amount of protein in the urine. Stat ultrasound Doppler of the bilateral lower extremity to rule out DVT was ordered. She is at high risk due to low albumin and nephrotic syndrome and may need to go back on Xarelto.

## 4. Discussion

Due to the concurrent lupus nephritis diagnosis and the knowledge that lupus affects all systems of the body, the concerns of lupus pneumonitis were initially not considered. Further, given the fact that much of the therapy for lupus is common for all subtypes, and many patients (occasionally) have involvement of multiorgan systems, the nephritis therapy may have been considered part of a pulmonary-renal syndrome as can be seen in lupus. Fortunately, in this case, the pneumonitis variant was considered.

The treatment of lupus may include high-dose steroids, antimalarials, anti-inflammatories, and immunosuppressants, e.g., calcineurins. Treatment of the systemic acute respiratory syndrome (SARS) virus, COVID-19, also includes steroids and anti-inflammatories. Fortunately, the recognition of the pneumonitis variant of lupus enabled additional lung scans and the detection of coronavirus pneumonitis, suggesting lupus pneumonitis masks coronavirus.

## 5. Conclusions

We discuss recommendations for expanded guidelines. Lupus patients, especially those with pulmonary involvement, should be screened for SARS (including COVID-19) to ensure the disease, nor the therapy, is masking the virus, especially when immunosuppressant therapy is considered. In this case, coronavirus pneumonitis can mimic, or present as, lupus pneumonitis. Pairing this report with a previously reported case [14], we propose that lupus patients, even when asymptomatic, be tested for COVID-19 prior to initiating immunosuppressive therapy. Specifically, patients with lupus that develop pneumonitis and fail to respond rapidly to conventional treatments should be regarded with a high index of suspicion for coronavirus.

## Data Availability

Data are available upon request from the authors, according to HIPPA regulations.

## Abbreviations

ANA: Antinuclear antibodies
bid: Twice daily
BMI: Body mass index
BP: Blood pressure
Bpm: Beats per minute
BUN: Blood urea nitrogen
COVID-19: Coronavirus or SARS-CoV-2
CT: Computed tomography
DNA: Deoxyribonucleic acid
DVT: Deep vein thrombosis
ESR: Erythrocyte sedimentation rate
GFR: Glomerular filtration rate
HR: Heart rate
IgG, IgA, IgM: Immunoglobulins
prn: As needed
qd: Once a day
RNP: Ribonucleoprotein
SLE: Systemic lupus erythematosus
SSA: Sjogren's syndrome antibodies
Th1: T helper 1
Th2: T helper 2
tid: Three times a day.
